# Altered Connectivity of the Anterior Cingulate and the Posterior Superior Temporal Gyrus in a Longitudinal Study of Later-life Depression

**DOI:** 10.3389/fnagi.2018.00031

**Published:** 2018-02-08

**Authors:** Kenichiro Harada, Toshikazu Ikuta, Mami Nakashima, Toshio Watanuki, Masako Hirotsu, Toshio Matsubara, Hirotaka Yamagata, Yoshifumi Watanabe, Koji Matsuo

**Affiliations:** ^1^Division of Neuropsychiatry, Department of Neuroscience, Yamaguchi University Graduate School of Medicine, Ube, Japan; ^2^Department of Communication Sciences and Disorders, School of Applied Sciences, University of Mississippi, Jackson, MS, United States; ^3^Nagato-Ichinomiya Hospital, Shimonoseki, Japan; ^4^Health Administration Center, Yamaguchi University Organization for University Education, Yamaguchi, Japan

**Keywords:** cingulate cortex, connectivity, gray matter volume, late-life depression, magnetic resonance imaging, resting state functional magnetic resonance imaging, superior temporal gyrus, white matter integrity

## Abstract

Patients with later-life depression (LLD) show abnormal gray matter (GM) volume, white matter (WM) integrity and functional connectivity in the anterior cingulate cortex (ACC) and posterior superior temporal gyrus (pSTG), but it remains unclear whether these abnormalities persist over time. We examined whether structural and functional abnormalities in these two regions are present within the same subjects during depressed vs. remitted phases. Sixteen patients with LLD and 30 healthy subjects were studied over a period of 1.5 years. Brain images obtained with a 3-Tesla magnetic resonance imaging (MRI) system were analyzed by voxel-based morphometry of the GM volume, and diffusion tensor imaging (DTI) and resting-state functional MRI were used to assess ACC–pSTG connectivity. Patients with LLD in the depressed and remitted phases showed significantly smaller GM volume in the left ACC and left pSTG than healthy subjects. Both patients with LLD in the depressed and remitted phases had significantly higher diffusivities in the WM tract of the left ACC–pSTG than healthy subjects. Remitted patients with LLD showed lower functional ACC–pSTG connectivity compared to healthy subjects. No difference was found in the two regions between depressed and remitted patients in GM volume, structural or functional connectivity. Functional ACC–pSTG connectivity was positively correlated with lower global function during remission. Our preliminary data show that structural and functional abnormalities of the ACC and pSTG occur during LLD remission. Our findings tentatively reveal the brain pathophysiology involved in LLD and may aid in developing neuroanatomical biomarkers for this condition.

## Introduction

Accumulated evidence from structural neuroimaging studies suggests that patients with later-life depression (LLD) show abnormal gray matter (GM) and white matter (WM) brain structures. Patients with LLD have a significantly smaller GM volume in the anterior cingulate cortex (ACC), superior temporal gyrus (STG), middle temporal gyrus, inferior frontal, orbitofrontal cortex, insula, hippocampus, amygdala and putamen than healthy individuals (Andreescu et al., 2008; Sexton et al., 2013; Du et al., 2014; Smagula and Aizenstein, 2016). In terms of WM structures, patients with LLD showed abnormal connectivity in the cingulum, uncinate fasciculus (Charlton et al., 2015), temporal lobe (Charlton et al., 2015), superior longitudinal fasciculus, superior frontal gyrus, corpus callosum (Reppermund et al., 2014) and parahippocampal gyrus (Guo et al., 2014). In our previous study, patients with LLD showed smaller GM volumes in the ACC, posterior STG (pSTG), and orbitofrontal cortex and abnormal WM integrity of the uncinate fasciculus, in comparison to healthy subjects (Harada et al., 2016). However, it remains unclear whether abnormalities of GM volume and WM connectivity are progressive or consistent over time.

Longitudinal neuroimaging studies of individuals with LLD have begun to address this issue. Measured over a 2-year period, the GM volume in patients with LLD a showed greater reduction in left hippocampal GM volume than in non-depressed subjects (Steffens et al., 2011). Although there is some evidence of change in WM intensities in patients with LLD over the long-term (Chen et al., 2006; Teodorczuk et al., 2007; Firbank et al., 2012), we are aware of only one longitudinal study of structural WM connectivity in patients with LLD to date (Khalaf et al., 2015). That study found no significant differences in the fractional anisotropy of WM tracts between LLD remitters and non-remitters over a 12-week antidepressant-treatment period (Khalaf et al., 2015).

While there is evidence of abnormal functional connectivity in resting state functional magnetic resonance imaging (rs-fMRI) studies in patients with LLD, the results remain inconclusive. Patients with LLD showed decreased, increased and comparable functional connectivity in a variety of regions as compared to healthy subjects in cross-sectional (Liu et al., 2012; Sexton et al., 2012; Andreescu et al., 2013; Wang et al., 2015; Eyre et al., 2016) and longitudinal studies (Wu et al., 2011; Alexopoulos et al., 2012; Andreescu et al., 2013). Functional connectivity of regions in the default-mode network has also been well investigated in LLD (Wu et al., 2011; Alexopoulos et al., 2012; Liu et al., 2012; Sexton et al., 2012; Andreescu et al., 2013; Wang et al., 2015; Eyre et al., 2016). This network is thought to be an organized functional network of several brain regions that are active during the resting state and inhibited during the performance of active tasks (Raichle et al., 2001). It involves activity in the ACC, posterior cingulate cortex, precuneus, medial frontal cortex, inferior parietal and the pSTG nearby the temporoparietal junction area (Mars et al., 2012). The network also largely overlaps with the social cognitive network (Mars et al., 2012); for instance, the neural network of “Theory of Mind” involves the connectivity between the ACC and the pSTG/temporoparietal junction area (Abu-Akel and Shamay-Tsoory, 2011). We previously reported a reduced GM volume in the ACC and pSTG in patients with LLD (Harada et al., 2016). Thus, in the present study, we focused on these two regions.

The aim of the present study was to determine whether the GM volume of the ACC and pSTG, and structural and functional connectivity in the ACC–pSTG network, would be longitudinally altered during the depressed and remitted phases in patients with LLD. We hypothesized that, compared to healthy participants, patients with LLD would show a reduced GM volume in the ACC and pSTG, and reduced WM integrity and functional connectivity in the ACC–pSTG during the depressed phase. We also expected that these structural and functional abnormalities would recover after remission.

## Materials and Methods

### Participants

This study was carried out in accordance with the latest version of the Declaration of Helsinki. The Institutional Review Board of Yamaguchi University Hospital approved this study. Written informed consent was obtained from all participants after providing them with a complete description of the study.

We studied 46 participants, including 16 patients who met the criteria of the Diagnostic and Statistical Manual of Mental Disorders, 4th Edition, Text Revision (American Psychiatric Association, 2000), for major depressive disorder, and 30 healthy subjects (Table 1). Patients with LLD and healthy subjects were not significantly different in terms of sex distribution, age, or years of education. Patients were recruited from Yamaguchi University Hospital and referred by clinics and hospitals in the area. Healthy participants were recruited by word-of-mouth or by advertising in the community. Patients were diagnosed and healthy subjects were screened based on clinical interviews and the Mini International Neuropsychiatric Interview (M.I.N.I., Japanese version 5.0.0; Otsubo et al., 2005). We confirmed the diagnoses in case conferences and laboratory meetings with distinguished clinical psychiatrists and trained research psychiatrists of our University.

**Table 1:** Demographic and clinical characteristics of the participants.

	LLD (*n* = 16)		Healthy (*n* = 30)
	Depressed	Remitted
Age (years)	56 [53.3–65.5]	58 [54–67]	58 [54–63.5]
Sex (Female %)	10 (62.5%)		19 (63.3%)
Education (years)	13 [12–15.8]		14 [12–16]
MMSE	28.5 [26.3–30]		30 [28.8–30]
Age of onset (years)	51 [33.5–59.8]		-
Duration (years)	7.5 [2.8–22.8]	9.3 [4.9–24.6]	-
Number of episode	2 [2–2]		-
SIGH-D^a,b^	20 [19–22.8]	2 [0.3–4.8]	1 [0–1]
GAF^a,b^	51 [51–60]	89.5 [81.3–90]	90 [90–95]
IMP-eq (mg)	143.8 [81.3–253.1]	200 [81.3–309.4]	-

*Data represent median [inter-quartile range]. LLD, later-life depression; MMSE, Mini-mental state examination; SIGH-D, Structured Interview Guide for the Hamilton Rating for Depression Scale; GAF, Global Assessment of Functioning; IMP-eq., Imipramine equivalent. ^a^Depressed vs. Remitted; *p* < 0.05 by Wilcoxon signed ranks test. ^b^Depressed vs. Healthy; *p* < 0.05 by Mann-Whitney *U* test*.

Some of the subjects participated in our previous study (Harada et al., 2016). Patients with a history of or current substance abuse/dependence and other psychotic illnesses, as assessed by the M.I.N.I. were excluded from the study. Using interviews, blood tests and physical examinations, subjects with endocrinological disease, head trauma, neurological disease, a family history of hereditary neurological disorders, or other medical conditions (i.e., hypertension, diabetes, active liver disease, kidney problems, or respiratory problems) were excluded from the study. Two patients had comorbid panic disorder without agoraphobia. Fourteen patients were taking medication at the time of study participation. Seven patients were taking only antidepressants; five were taking antidepressants and antipsychotics; one was taking an antidepressant and a mood stabilizer; and one was taking an antidepressant, an antipsychotic, and a mood stabilizer. Of the remaining two patients, one was drug-naïve, and the other had been medication-free for at least 4 years. The medication load was assessed using imipramine-equivalent doses (Inada and Inagaki, 2015). The imipramine-equivalent dosage did not differ significantly between the depressed and remitted phase in patients with LLD. Healthy subjects were excluded if they had an immediate family member with a psychiatric disorder. Current mood states were evaluated using the Structured Interview Guide for the Hamilton Depression Rating Scale (SIGH-D; Williams, 1988). Any participant who was left-handed or ambidextrous was excluded from the analysis (Oldfield, 1971). The Global Assessment of Functioning scale (GAF, 2000) was used to assess social functioning. None of the participants were demented at the time of study participation, according to a Mini-Mental State Examination (MMSE; Folstein et al., 1975) and clinical interview. Patients with LLD underwent clinical evaluations and MRI examination during the depressed and remitted phases. Remission was defined using the DSM-IV criteria for full remission, with a specifier depressive episode, and with a score < 8 on the SIGH-D. The median [inter-quartile range] of duration from the depressed phase to recovery phase was 514.5 [145.2–877.3] days.

### Magnetic Resonance Imaging

Brain images were collected on a Siemens 3-Tesla MRI system (Siemens Medical System, Skyra, Erlangen, Germany) using T1-weighted imaging, diffusion tensor imaging (DTI) with eigenvalues, and rs-fMRI. The T1-weighted parameters were as follows: field of view, 270 mm; view matrix, 256 × 256; repetition time, 2300 ms; echo time, 2.95 ms; flip angle, 9°; and slice thickness, 1.2 mm. The T2-weighted parameters were as follows: field of view, 240 mm; view matrix, 394 × 512; repetition time, 5000 ms; echo time, 87 ms; flip angle, 150°; and slice thickness, 6 mm. The DTI parameters were as follows: field of view, 235 mm; view matrix, 100 × 100; repetition time, 8700 ms; echo time, 88 ms; flip angle, 90°; and slice thickness, 2.4 mm. Two *b* values were used; one image was acquired at 0 s/mm^2^ (no diffusion weighting) and 29 noncoplanar images were acquired at 1000 s/mm^2^ (diffusion-weighted *b* value). The rs-fMRI parameters were as follows: field of view, 220 mm; view matrix, 64 × 64; repetition time, 2500 ms; echo time, 30 ms; flip angle, 80º, slice thickness, 4 mm. MR images were manually checked for quality before the analyses and abnormal findings were evaluated by radiologists who were blinded to the participants’ diagnoses.

### Image Analysis

#### GM Volume

Image preprocessing was performed using VBM8^1^ in the SPM8 software (Wellcome Department of Imaging Neuroscience, London, UK) running under Matlab R2015b (MathWorks, Natick, MA, USA). All original images were manually aligned on the anterior-posterior commissure line. T1-weighted images were segmented and imported into a format that could be used by the VBM8 algorithm. The segmentation procedure automatically removed non-brain tissues, including the scalp, skull and dural venous sinuses. The segmented images were normalized to Montreal Neurological Institute (MNI) space using the template assembled in VBM8, and were smoothed with an 8-mm Gaussian filter.

#### WM Connectivity

DTI scans were processed using the Functional MRI of the Brain Software Library (FSL version 5.1; Oxford, UK^2^). Eddy-current distortions and head displacements were adjusted through affine registration of the 31 diffusion volumes to the first b0 volume using FSL’s Linear Registration Tool. The b-vector table (i.e., gradient directions) for each participant was then aligned to the rotation parameters of this linear correction. Non-brain tissue was removed using FSL’s Brain Extraction Tool. Fractional anisotropy, as well as axial, radial, and mean diffusivities, were then estimated at each voxel of the brain by fitting a diffusion tensor model to the raw diffusion data using weighted least-squares in FSL’s Diffusion Toolbox.

The local (i.e., within-voxel) probability density functions of the principal diffusion direction were estimated using Markov Chain Monte Carlo sampling in FSL’s Bedpostx tool (Behrens et al., 2007). A spatial probability density function across voxels was then estimated based on these local probability density functions using FSL’s Probtrackx tool (Behrens et al., 2007), in which 5000 samples were taken for each input voxel with a 0.2-curvature threshold, 0.5-mm step length, and 2000 steps per sample.

A 5-mm sphere was created at the pSTG peak in the VBM finding (MNI: −46.5, −61.5, 18) in MNI 1-mm space. The ACC was defined using Freesurfer 5.3.0 (Fischl et al., 1999, 2002) on MNI 1-mm brain space. Probabilistic tractography was used to segment the WM structure between the pSTG sphere and the ACC. The mean of the axial, radial, and mean diffusivities, as well as the fractional anisotropy was calculated within the WM tract for each individual. Probabilistic tractography failed to segment the WM tract of nine healthy subjects and three patients with LLD in remission.

#### Functional Connectivity

Script libraries (fcon) from the 1000 Functional Connectomes Project^3^ (Biswal et al., 2010) were used for preprocessing and region of interest (ROI) analyses. Resting state images were first motion-corrected and spatially smoothed using a 6-mm full-width at half-maximum Gaussian kernel. The structural T1 images were individually registered to the MNI152 2-mm brain. Using this registration, 12 affine parameters were created between the rs-fMRI volume and MNI152 with a 2-mm space, such that a seed ROI could later be registered to each individual rs-fMRI space. The time series were band-pass filtered (between 0.005 Hz and 0.1 Hz), and each resting state volume was regressed by WM and cerebrospinal fluid signal fluctuations as well as the six motion parameters. For ROI connectivity analysis, ROIs were determined in the MNI152 2-mm space. The same pSTG and ACC ROIs from the WM analysis were registered to the MNI 2-mm space. Between these two ROIs, a Pearson’s product moment correlation coefficient was calculated for each subject and then transformed to the Z standard score.

### Statistical Analysis

#### GM Volume

We analyzed the imaging data with SPM8 software, which implements a general linear model. We examined the VBM analysis of the whole brain and of an ROI. The left ACC and bilateral STG were defined as an ROI according to our previous study (Harada et al., 2016). SPM8 was used to assess the difference in GM volume between patients with LLD (depressed and remitted) and healthy subjects, using a two-sample *t*-test, with age, sex, years of education, and total brain volume as covariates. Comparison between depressed patients with LLD and remitted patients with LLD was performed using a paired *t*-test in SPM8. A voxel-wise *t*-test was performed with the threshold set at *p* < 0.05 corrected by family-wise error (*p*_FWE_) in SPM8. We anatomically identified the brain regions using automated anatomical labeling (Tzourio-Mazoyer et al., 2002) via the Wake Forest University Pick Atlas (version 3.04^4^; Maldjian et al., 2004) and the Atlas of the Human Brain (Mai et al., 2008). All results are presented in MNI coordinates.

#### WM Connectivity

We compared the mean diffusivity, radial diffusivity, axial diffusivity and fractional anisotropy values of the left ACC–pSTG WM tract between depressed patients with LLD and healthy subjects and between remitted patients with LLD and healthy subjects, using a Mann-Whitney *U* test. We also compared these values of the WM tract between depressed and remitted patients with LLD using the Wilcoxon signed-rank test. SPSS Statistics version 20 for Windows (IBM, Chicago, IL, USA) was used for statistical analysis.

#### Functional Connectivity

To test functional connectivity changes and differences between groups, Z scores of functional connectivity of the ACC–pSTG tract were examined and compared between depressed patients with LLD and healthy subjects, and between remitted patients with LLD and healthy subjects, using the Mann-Whitney *U* test, and between depressed and remitted patients with LLD using Wilcoxon’s signed-rank test.

#### Multi-modal and Clinical Associations

Spearman’s rank correlation coefficients were tested to determine the association between clinical variables and regions in patients with LLD when statistical analysis showed significance between patients with LLD and healthy participants. The clinical variables included the duration of illness, the duration of recovery, the number of episodes, imipramine equivalent and the total SIGH-D and GAF scores. We also examined the correlation between the subscore of “feelings of guilt” in the SIGH-D and the results of MRI analyses, because this feeling represents the core feature of cognitive disturbance in depression and is associated with the ACC and pSTG for mood processing and with the default-mode network (Basile et al., 2011; Andrews-Hanna et al., 2014; Hamilton et al., 2015; details in “Discussion” section). Statistical significance was set at *p* < 0.05.

## Results

### GM Volume

**Figure 1:**
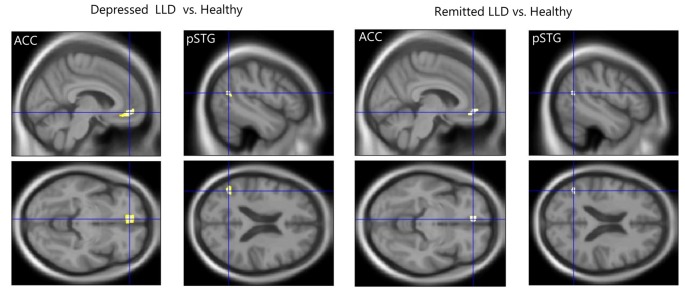
Reduced gray matter (GM) volume in patients with later-life depression (LLD). Patients with acute LLD showed significantly smaller GM volumes in the left anterior cingulate cortex (ACC) and posterior superior temporal gyrus (pSTG) than did healthy subjects. Patients with LLD in remission showed significantly smaller GM volumes in the left ACC and pSTG than healthy participants. The cross-hairs mark the coordinates of the voxel of maximal statistical significance.

The whole brain analysis demonstrated that depressed patients with LLD showed smaller GM volumes in the left pSTG than healthy subjects (the coordinates of the voxel of maximum, *x* = −47, *y* = −61, *z* = 18, *T* = 6.55, *k* = 77, *P*_FWE_ < 0.001). No other regional GM volume was significantly different in the whole brain analysis. There was no significant difference in the GM volume in the whole brain analysis between remitted patients with LLD and healthy subjects, or between patients with LLD in the depressed and remitted phases. For the ROI analysis, depressed patients with LLD had significantly smaller GM volumes in the left ACC (the coordinates of the voxel of maximum, *x* = −6, *y* = 45, *z* = −9, *T* = 5.25, *k* = 440, *P*_FWE_ < 0.001) and left pSTG (*x* = −47, *y* = −61, *z* = 18, *T* = 6.55, *k* = 186, *P*_FWE_ < 0.001) than healthy subjects. Remitted patients with LLD also showed significantly smaller GM volumes in the left ACC (*x* = −6, *y* = 44, *z* = −11, *T* = 4.05, *k* = 162, *P*_FWE_ = 0.010) and left pSTG (*x* = −47, *y* = −61, *z* = 18, *T* = 5.40, *k* = 93, *P*_FWE_ = 0.003) than healthy participants (Figure 1). There was no significant difference in the GM volume of the two regions between patients with LLD in the depressed and remitted phases.

### WM Connectivity

**Figure 2:**
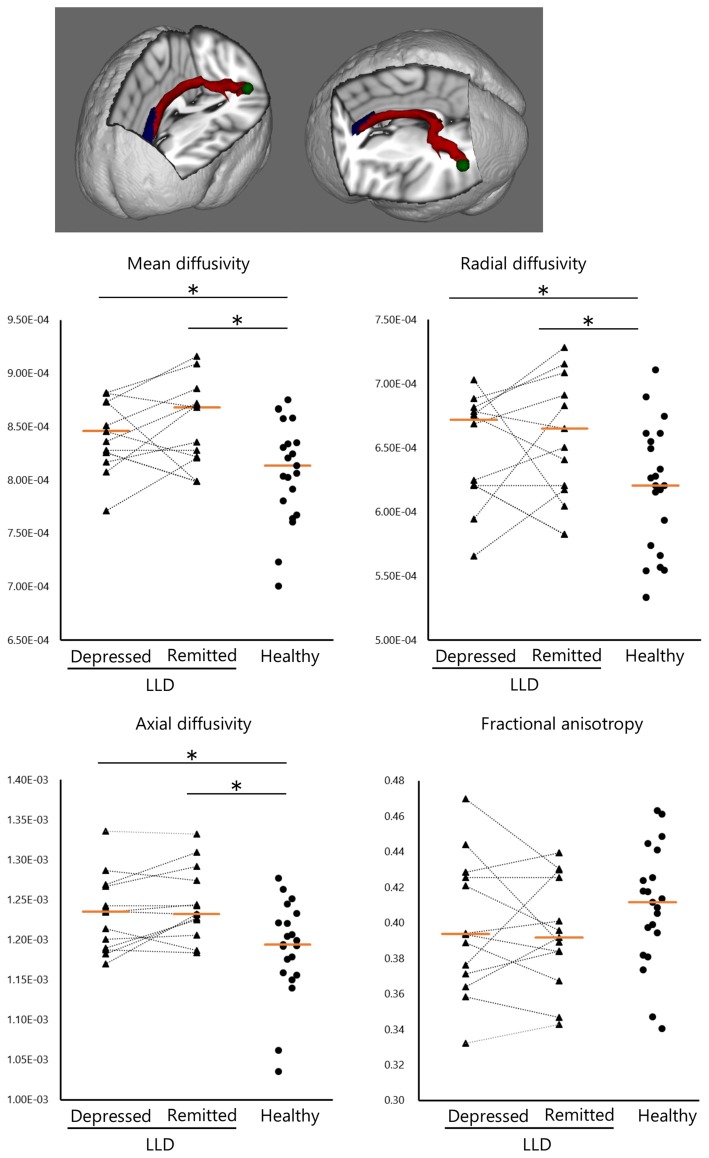
Structural white matter (WM) connectivity between the left anterior cingulate and posterior superior temporal gyrus (ACC–pSTG). Patients with LLD (*n* = 13) in the depressed and remitted phases demonstrated significantly higher WM connectivity in the ACC–pSTG region than did healthy subjects (*n* = 21), in terms of mean, radial and axial diffusivity, but not in fractional anisotropy. Tangerine bars represent the median value of Z-score for each diffusivity. Y-axis represents z-score. *p < 0.05 by Mann-Whitney *U* test or Wilcoxon signed ranks test.

We found that depressed patients with LLD showed significantly higher mean diffusivity, radial diffusivity, and axial diffusivity, but not fractional anisotropy, in the WM tract of the left ACC–pSTG than healthy subjects (mean diffusivity, *U* = 251, *P* = 0.01; radial diffusivity, *U* = 239, *P* = 0.03; axial diffusivity, *U* = 242, *P* = 0.02; fractional anisotropy, *U* = 140, *P* = 0.40; Figure 2). Similarly, remitted patients with LLD had significantly higher scores on those parameters than healthy participants (mean diffusivity, *U* = 210, *P* < 0.01; radial diffusivity, *U* = 201, *P* = 0.02; axial diffusivity, *U* = 101, *P* < 0.01; fractional anisotropy, *U* = 101, *P* = 0.22). There was no statistically significant difference between depressed and remitted patients with LLD in any parameters of WM tracts (mean diffusivity, *Z* = −1.02, *P* = 0.31; radial diffusivity, *Z* = −0.47, *P* = 0.64; axial diffusivity, *Z* = −1.49, *P* = 0.14; fractional anisotropy, *Z* = −0.39, *P* = 0.70).

### Functional Connectivity

**Figure 3:**
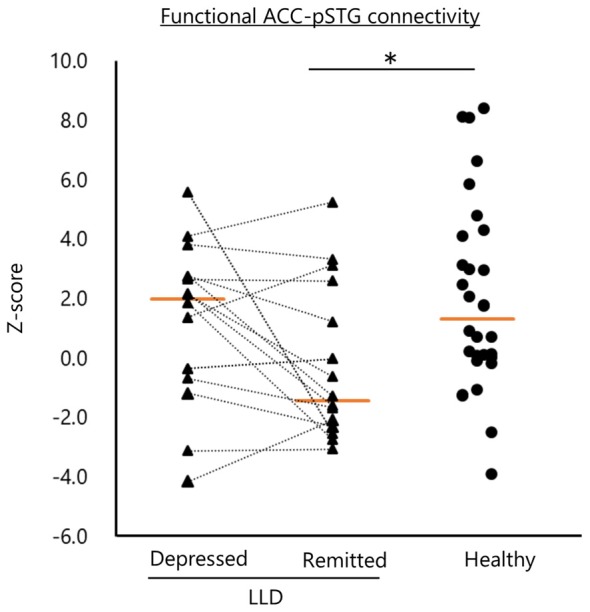
Functional connectivity of the left anterior cingulate and posterior superior temporal gyrus (ACC–pSTG). Patients with remitted LLD (*n* = 16) showed lower functional connectivity in the left ACC–pSTG than healthy subjects (*n* = 30). Tangerine bars represent the median value of Z-score for each group. *p < 0.05 by Mann-Whitney *U* test.

Remitted patients with LLD showed lower functional connectivity in the left ACC–pSTG than did healthy subjects (*U* = 127, *P* < 0.01; Figure 3). The functional connectivity was not statistically significantly different between depressed and remitted patients with LLD or between depressed patients with LLD and healthy subjects.

### Multi-modal and Clinical Associations

**Figure 4:**
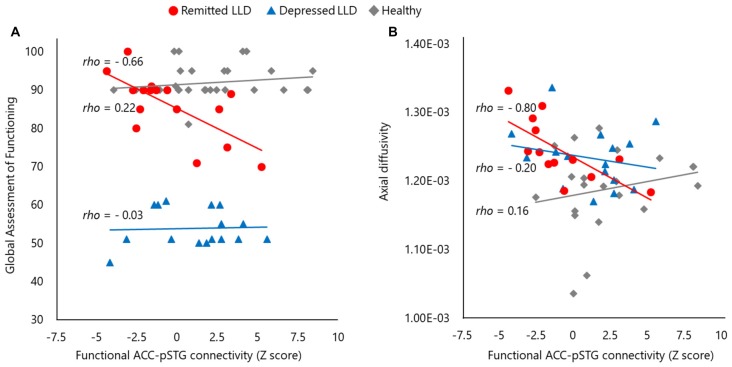
The scatter plots of between functional and structural connectivity and the global functioning. Functional connectivity of anterior cingulate and posterior superior temporal gyrus pSTG (ACC–pSTG) showed a significantly inverse correlation in the score of global assessment of functioning **(A)** and structural ACC–pSTG connectivity **(B)** in patients with LLD in the remitted phase (red circle), but not in patients with LLD in the depresses phase (blue triangle) or healthy participants (gray diamond).

There was no significant correlation between clinical variables, including “feelings of guilt” in the SIGH-D, and the structural or functional MRI findings in depressed patients with LLD and healthy subjects. The functional connectivity of the ACC–pSTG was significantly and inversely correlated with the GAF score (*rho* = −0.66, *p* < 0.01) and the axial diffusivity of the ACC–pSTG WM tract (*rho* = −0.80, *p* < 0.01) in patients with LLD in the remitted phase, but not in patients with LLD in the depressed phase or healthy subjects (Figure 4). These two results remained statistically significant after Bonferroni correction. The other clinical variables, including medication load, were not significantly correlated with any result of structural or functional MRI in remitted patients with LLD.

To verify the significance of these results (Figure 4), we performed an exploration of the differences in the two correlations between the groups, using Fisher’s *Z* test. In terms of functional connectivity and GAF correlation, there was a significant difference between the remitted phase (*Z* = −0.80) and the depressed phase (*Z* = 0.03) in patients with LLD (*Z* = 2.12, *p* = 0.03) and between remitted patients and healthy subjects (*Z* = 0.21; *Z* = 3.03, *p* < 0.01), but not between depressed patients with LLD and healthy subjects (*Z* = 0.56, *p* = 0.58). In terms of functional connectivity and axial diffusivity correlation, there was a significant difference between the remitted phase (*Z* = −1.09) and depressed phase (*Z* = −0.21) in patients with LLD (*Z* = 2.25, *p* = 0.02), and between remitted patients and healthy subjects (*Z* = −0.12; *Z* = 2.65, *p* < 0.01), but not between depressed patients with LLD and healthy subjects (*Z* = 0.22, *p* = 0.83). These results suggested that the two observed statistically significant correlations were valid in patients with LLD in the remitted phase.

## Discussion

Although this is a preliminary study, no other longitudinal investigation of structural and functional connectivity abnormalities in patients with LLD has been reported to date. Acutely depressed patients with LLD, relative to healthy subjects, had a smaller GM volume in the ACC and pSTG, higher WM connectivity, and comparable functional connectivity between the two regions. These GM and WM anomalies remained abnormal after remission in patients with LLD, while the functional connectivity declined in patients with LLD during remission relative to healthy subjects. Furthermore, ACC–pSTG functional connectivity was associated with structural connectivity of the ACC–pSTG and global functioning in the remitted phase. The present results suggest that: (1) structural abnormalities in the ACC and pSTG do not appear to be linked to mood state; (2) functional abnormality of the ACC–pSTG connection is associated with functional outcome; and (3) GM volume and structural and functional connectivity of ACC and pSTG are, to some extent, linked to each other. These tentative findings indicate that the ACC–pSTG is both structurally and functionally involved in the pathophysiology of LLD.

The ACC connects structurally and functionally with a variety of brain areas, including the lateral prefrontal cortex, orbitofrontal cortex, parietal cortex, amygdala, STG, nucleus accumbens, hypothalamus, insula, raphe nucleus and hippocampus (Morecraft and Van Hoesen, 1992; Devinsky et al., 1995; Margulies et al., 2007; Ikuta et al., 2017). The ACC plays a role in processing stimulation, error detection, prediction of the problem, incentive and modulation of emotional reaction (Bush et al., 2000; Gasquoine, 2013). Substantial neuroimaging studies have shown that the ACC is deeply involved in depression. One review of preclinical and clinical studies has suggested that a neural network that includes orbital and medial prefrontal cortex regions, such as the ACC, the limbic system, the STG, and the temporal lobe is associated with depression (Price and Drevets, 2010). Deep brain stimulation of the subgenual ACC was effective for biological treatment of patients with treatment-resistant depression (Mayberg et al., 2005).

However, the evidence for reduced GM volume of ACC in patients with LLD is inconsistent (Lavretsky et al., 2007; Yuan et al., 2008; Weber et al., 2010). A reduced GM volume of the ACC was observed during both depressed and remitted phases in the current as well as a prior study (Harada et al., 2016). The reasons for inconsistency across studies of LLD may be related to methodological differences, such as the mood state, the mean age of participants, the number of episodes, and the duration of illness. Further studies controlling for these background demographics are needed to obtain conclusive data regarding abnormal GM volumes in the ACC in patients with LLD.

The pSTG is a part of the temporoparietal junction area (Abu-Akel and Shamay-Tsoory, 2011). This area plays a crucial role in integrating information from both the external environment as well as from within the body, and in collecting and processing all of this information (Abu-Akel and Shamay-Tsoory, 2011). The area is also known to be a key player in cognitive and affective theory of mind (Saxe and Kanwisher, 2003; Corradi-Dell’Acqua et al., 2014), such as false belief (Apperly et al., 2004; Aichhorn et al., 2009). Structured studies have shown reduced GM volume and density of the STG in mood disorders (Nugent et al., 2006; Abe et al., 2010; Peng et al., 2011). Our results support these findings.

The present study demonstrated no difference in the GM volume in the ACC or pSTG in the same patients during depressed and remitted phases. To our knowledge, no previous longitudinal neuroimaging study has measured such regional GM volumes in patients with LLD. Steffens et al. (2011) have demonstrated that patients with LLD had a reduced hippocampal GM volume over a 2-year period, while non-depressed subjects did not, and the reduction of hippocampal GM volumes was associated with a decline in the MMSE for 2–2.5 years in patients with LLD. They also measured hippocampal GM volume using a manual tracing method and did not examine the GM volume of other regions, while we performed a whole brain VBM analysis. Their study did not show whether patients with LLD were remitted when the GM volume was measured for a second time. They determined the MMSE score at the baseline and at a second point, while we obtained the MMSE score in the remitted phase, because depressed patients often demonstrate psychomotor retardation, which may affect the MMSE score. Thus, it was unknown whether the patients showed cognitive decline from the baseline to the second assessment point. The sample size in total was 162 in their study, vs. the 46 participants in the current study. These methodological differences between the two studies make it difficult to interpret the differences in results. More longitudinal studies with large samples, controlling for background demographics, are required to obtain conclusive data on the abnormal GM volumes in patients with LLD.

In terms of the ACC–pSTG connectivity, the current study showed structural and functional abnormalities in patients with LLD, and these abnormalities were associated with clinical manifestations. The pSTG and ACC are anatomically and functionally connected in the monkey (Yukie and Shibata, 2009) and humans (Margulies et al., 2007). The two regions are involved in the default-mode network and social cognitive network (Margulies et al., 2007; Menon, 2011). Reviews of rs-fMRI studies of depression have shown abnormal functional connectivity between the two regions (Kaiser et al., 2015; Mulders et al., 2015). Although we are not aware of a study that directly examined functional connectivity of the ACC–pSTG in depression, our results contribute to such evidence.

The WM ACC–pSTG connectivity showed state-independent abnormality in patients with LLD: the axial, radial, and mean diffusivities of the ACC–pSTG tract were higher in patients with LLD. Fractional anisotropy of the ACC–pSTG did not show a significant difference between patients with LLD and healthy subjects. This lack of difference in fractional anisotropy is consistent with our finding that both axial and radial diffusivities are higher in the patients with LLD. Fractional anisotropy is an estimate of the superlativeness of axial diffusivity as compared to the secondary and tertiary diffusivities, radial diffusivity is the mean of the secondary and tertiary diffusivities, while mean diffusivity (also known as the apparent diffusion coefficient) is the mean of the axial, secondary, and tertiary diffusivities. The lack of difference in fractional anisotropy and the higher mean diffusivity in the LLD group is the result of the higher axial and radial diffusivities. Therefore, higher mean diffusivity is the main structural connectivity finding of the current study. Increased mean diffusivity has been repeatedly reported in multiple sclerosis, especially during the acute phase (Christiansen et al., 1993; Tievsky et al., 1999; Werring et al., 2000; Rosso et al., 2006). Our results may imply de-myelinated WM tissue in LLD.

We cautiously propose the following speculative model to describe the MRI results, based on the above discussion. Patients with LLD are structurally and functionally vulnerable in the ACC–pSTG during the asymptomatic state. This may involve maintaining a balance with low-grade ACC–pSTG activity. In an acutely depressed state, the functional ACC–pSTG connectivity would increase to compensate for the weak neural activity of the small GM volume in these two regions, in order to regulate negative ruminative mood and cognition. Subsequently, the functional ACC–pSTG connectivity during the depressed phase would be relatively overactive in these patients, although seeming to be in a normal range as compared to healthy subjects. Then, this hyper-functional connectivity would induce an imbalance between structure and function of the ACC–pSTG, leading to inappropriate mood and cognitive processing, and may result in symptomatic manifestation. However, the overactivation of STG in an rs-fMRI study was observed in depressive patients with a history of suicide attempt, as compared to those without such a history and healthy subjects (Cao et al., 2016). The study also showed that the overactivation was also associated with abnormal impulsivity in suicide attempters. The investigators stated a similar interpretation of the results, together with the finding of a small GM volume in the STG in suicide attempters in another study (Pan et al., 2015). However, this model is speculative, because the functional connectivity was not different between the depressed and remitted stages in patients with LLD, and no other functional or structural connectivity was investigated. To test such a model, substantial multi-modal neuroimaging studies for depression and depression-like animal model studies would be required to elucidate the neural mechanism of mood regulation and cognitive processing. A potential molecular mechanism of the structural and functional vulnerability in LLD may be the effect of inflammation on dysfunctional processing. Some evidence has suggested that structural abnormalities, e.g., vascular depression and hypoperfusion of the brain, were observed in patients with LLD (Alexopoulos et al., 1997; Matsuo et al., 2005); these abnormalities may trigger microglial activation and subsequent neuroinflammation, and this abnormal neuroinflammation processing may lead to demyelination and neurodegeneration (Popa-Wagner et al., 2014; Becker, 2016). To address this hypothesis, inflammatory biomarkers, including interleukin 6 and 8, interferon-alpha, IL-6, IL-8, IFN-alpha and TNF-alpha, together with measurement of structural and functional neuroimaging, would be necessary.

There are some limitations to this study: first, the sample size was small. Thus, statistical power would be somewhat limited, although we applied non-parametric statistical tests. Second, medication may have affected the MRI results, although the effect of medication was inconclusive in patients with mood disorders in meta-analytical and review studies (Koolschijn et al., 2009; Bora et al., 2012; Matsuo et al., 2012). Although the GM volume of the ACC and pSTG, and structural and functional connectivity of the two regions were not associated with medication load in the present study, we cannot exclude the possibility that medication could have masked the results. Third, as we conducted a seed-based approach in the ACC and pSTG, other regions relevant to mood and cognitive processing in LLD may have indirectly influenced the results related to the functional connectivity of these two regions. Lastly, we did not perform MRI re-scanning in healthy subjects; thus it cannot be excluded that structural and functional changes in certain regions may have occurred in healthy subjects over the observation period, which may alter the conclusions of the current study.

In conclusion, the findings of the present preliminary study provide tentative evidence that structural and functional abnormalities of the ACC and pSTG are observed during remission of LLD. Structural connectivity of the ACC–pSTG was independent of mood state and functional ACC–pSTG connectivity was associated with functional outcome. Our findings reveal some insights into brain pathophysiology and may aid in developing neuroanatomical biomarkers for LLD.

## Author Contributions

KM conceived and designed the experiments. KH, MN, TW, MH, TM, HY and YW performed the experiments and collected the data. KH and TI analyzed MR data. KH, TI and KM discussed the results and wrote the article.

## Conflict of Interest Statement

TI has received speaker’s honoraria from Eli Lilly, Daiichi Sankyo and Dainippon Sumitomo. KM received honoraria from GlaxoSmithKline, Mochida Pharmaceutical, and Meiji Seika Pharma and research donations from Otsuka Pharmaceutical. YW received research donations from MSD, GlaxoSmithKline, Eli Lilly and Company, Yoshitomiyakuhin, Shinogi, Pfizer, Janssen Pharma, Meiji Seika Pharma, FujiFilum RI Pharma, Takeda Pharmaceutical, Astellas, Dainippon Sumitomo Pharma and Otsuka Pharmaceutical. The other authors declare that the research was conducted in the absence of any commercial or financial relationships that could be construed as a potential conflict of interest.

## Footnotes

^1^http://dbm.neuro.uni-jena.de/vbm/

^2^http://fsl.fmrib.ox.ac.uk/fsl

^3^http://www.nitrc.org/projects/fcon_1000

^4^http://fmri.wfubmc.edu/software/PickAtlas
